# Cholera returns to southern Vietnam in an outbreak associated with consuming unsafe water through iced tea: A matched case-control study

**DOI:** 10.1371/journal.pntd.0005490

**Published:** 2017-04-13

**Authors:** Thuong V. Nguyen, Quang D. Pham, Quoc K. Do, Tai T. Diep, Hung C. Phan, Thang V. Ho, Hong T. Do, Lan T. Phan, Huu N. Tran

**Affiliations:** 1 Pasteur Institute, Ho Chi Minh City, Vietnam; 2 Department for Disease Control and Prevention, Pasteur Institute, Ho Chi Minh City, Vietnam; 3 Department of Microbiology and Immunology, Pasteur Institute, Ho Chi Minh City, Vietnam; 4 Ben Tre Preventive Health Centre, Ben Tre, Vietnam; Johns Hopkins Bloomberg School of Public Health, UNITED STATES

## Abstract

**Background:**

After more than a decade of steadily declining notifications, the number of reported cholera cases has recently increased in Vietnam. We conducted a matched case-control study to investigate transmission of cholera during an outbreak in Ben Tre, southern Vietnam, and to explore the associated risk factors.

**Methodology/Principal findings:**

Sixty of 71 diarrheal patients confirmed to be infected with cholera by culture and diagnosed between May 9 and August 3, 2010 in Ben Tre were consecutively recruited as case-patients. Case-patients were matched 1:4 to controls by commune, sex, and 5-year age group. Risk factors for cholera were examined by multivariable conditional logistic regression. In addition, environmental samples from villages containing case-patients were taken to identify contamination of food and water sources. The regression indicated that drinking iced tea (adjusted odds ratio (aOR) = 8.40, 95% confidence interval (CI): 1.84–39.25), not always boiling drinking water (aOR = 2.62, 95% CI: 1.03–6.67), having the main source of water for use being close to a toilet (aOR = 4.36, 95% CI: 1.37–13.88), living with people who had acute diarrhea (aOR = 13.72, 95% CI: 2.77–67.97), and little or no education (aOR = 4.89, 95% CI: 1.18–20.19) were significantly associated with increased risk of cholera. In contrast, drinking stored rainwater (aOR = 0.17, 95% CI: 0.04–0.63), eating cooked seafood (aOR = 0.27, 95% CI: 0.10–0.73), and eating steamed vegetables (aOR = 0.22, 95% CI: 0.07–0.70) were protective against cholera. *Vibrio cholerae* O1 Ogawa carrying *ctxA* was found in two of twenty-five river water samples and one of six wastewater samples.

**Conclusions/Significance:**

The magnitude of the cholera outbreak in Ben Tre was lower than in other similar settings. This investigation identified several risk factors and underscored the importance of continued responses targeting cholera prevention in southern Vietnam. The association between drinking iced tea and cholera and the spread of *V*. *cholerae* O1, altered El Tor strains warrant further research. These findings might be affected by a number of limitations due to the inability to capture asymptomatic or mildly symptomatic infections, the possible underreporting of personal unhygienic behaviors, and the purposive selection of environmental samples.

## Author summary

Cholera is a highly contagious, acute diarrheal illness, which poses a profound health threat in many parts of the less developed world. The majority of cases are reported from Sub-Saharan Africa, South-East Asia, and the Americas (i.e., Haiti) where infections are primarily transmitted through ingestion of contaminated water. Today in the era of widely available rehydration therapies and antibiotics, deaths due to cholera are quite rare. Despite this, early detection of contaminated water sources is crucial for directing early interventions for curbing community-wide transmission. The authors found evidence linking an outbreak of cholera in southern Vietnam to consumption of unsafe water, especially drinking iced tea. This finding suggests the need for a water-monitoring system at ice-making plants. Further research is needed to confirm the biological link between iced tea consumption and cholera infection. Larger studies should also be conducted to understand the clinical consequences of infection with the new cholera agent (*V*. *cholerae* O1, altered El Tor strains).

## Introduction

Cholera is a highly contagious diarrheal disease, caused by infection of the Gram-negative bacterium *Vibrio cholerae* [1]. Areas with poverty, high population-density, poor sanitation, poor education levels, and lack of potable water are at risk for cholera outbreaks [2–4]. An estimated 1.3–4.0 million illnesses and 21,000–143,000 deaths are attributed directly to the disease, which is predominantly seen in Sub-Saharan Africa, South-East Asia, and the Americas (i.e., Haiti) [5]. Consumption of contaminated water is thought to be the main mode of transmission [6–8].

Prior to implementation of control measures, Vietnam suffered a disproportionate burden of cholera. For example, between 1979 and 1996, there were 56,050 reported cases of cholera and 1,272 deaths due to cholera in this country [9]. Oral cholera vaccination programs were introduced in highly endemic areas through a national expanded program of immunization in 1997 [10]. The program, in conjunction with improved personal hygiene and access to potable drinking water associated with both health promotion programs and economic growth, led to a substantial drop in the number of notified cholera cases [6, 10]. However, these gains have not been sustained. Resource constraints [10] as well as the modest vaccine effectiveness, estimated to be 76% during the outbreak and 50% after 3–5 years [11, 12], have jeopardized the long-term impact of this program.

There were a total of 3,646 cholera cases reported between 2006 and 2010 in Vietnam, which was nearly five times higher than the number of notification made during 2001–2005 (747 cases) [13]. Of concern, from both a clinical and public health perspective, is the emergence and transmission of *V*. *cholerae* O1, El Tor strains that produce the classical cholera toxin [14] and the circulation of strains genetically resistant to certain antibiotics [9, 15]. Outbreaks in Hanoi and neighboring regions in northern Vietnam in 2007–2008 have been linked to contaminated food, especially dog meat, raw vegetables, and raw pig/duck blood [11, 13]. However, the pathways for the transmission of cholera in other parts of the country have not yet been investigated.

In this article, we report a cholera outbreak in the Mekong Delta province of Ben Tre in 2010. We conducted a matched case-control study to identify risk factors for cholera infection, and examined environmental samples to identify contaminated food and water sources. These data are useful for informing targeted and effective responses to cholera outbreaks in Vietnam.

## Methods

### Study settings

**Fig 1:**
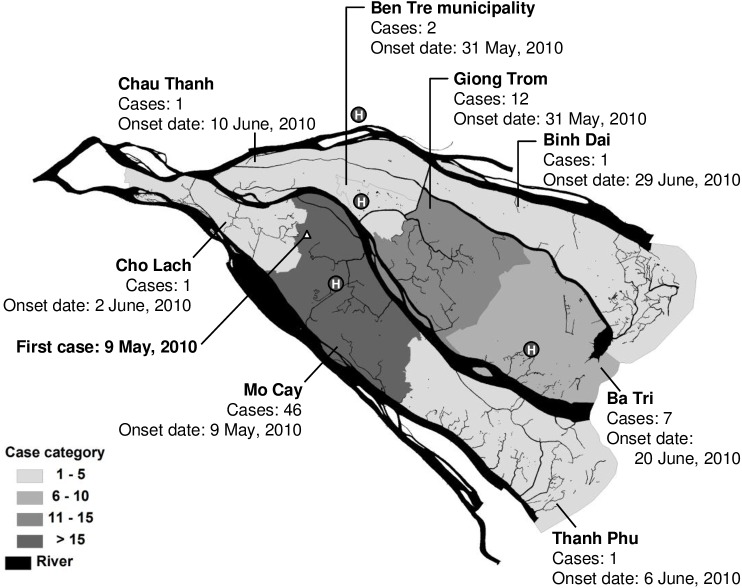
Map showing the location of 71 culture confirmed cholera cases in Ben Tre, Vietnam during May-August 2010. The triangle represents the first case of cholera identified in May 9, 2010 in Tan Phu Tay commune, northern Mo Cay district, Ben Tre province. Letter H inside a black circle represents an assigned cholera treatment center in this outbreak.

Ben Tre province is located in the Mekong Delta region of southern Vietnam. It has a population of 1.3 million people and a largely agricultural economy. This coastal province is about 1.25 meters above sea level and is almost surrounded by water (Fig 1). Due to low piped-water coverage, river water is generally used as one of the main sources of water among the population. The last mass oral cholera vaccine program was implemented in 2002, and no cholera cases had been reported since 2005 [10, 16]. However, in 2010, an outbreak of cholera occurred in the province, starting in Mo Cay town on May 9. A total of 71 cases were identified in the outbreak (Fig 1).

### Collection of food and water samples

We employed a purposive sampling technique to collect water samples from several types of water sources in Mo Cay and Giong Trom towns, where the first cholera cases were reported in this outbreak. They included 25 samples of river water obtained from the rivers closest to case-patients’ houses; six samples of indoor water, six wastewater samples, and two samples of drinking water in case-patients’ houses. In addition, 27 samples of fresh seafood were obtained from local markets where the case-patients lived.

### Case-control study

From June 4 through July 2, 2010, we conducted a matched case-control study with a target sample size of 60 case-patients and 240 controls. Because the outbreak occurred in a not well-defined population and required rapid investigation, the case-control study would be the most appropriate choice of study design to identify risk factors for cholera infection [17]. In this study, the matching by age groups, sex, and living areas was used [18]. The sample size of this study for 80% power at 5% level of significance was calculated on the basis of the estimated percentage of controls exposed to unsafe water (30%), the ratio of controls to cases (4), and an odds ratio (OR, 2.25) as per the method of Kelsey and colleagues [19]. The estimated magnitude of OR that we used to calculate the sample size came from the previous work of Hoge and his colleagues [8]. The smallest reported OR was selected to ensure that the study was large enough and had sufficient statistical power in identifying risk factors with an expected OR of 2.25 or higher.

During this outbreak, all patients with acute watery diarrhea who sought diagnosis and treatment for their disease at local health facilities were immediately transferred to the nearest cholera treatment center. In all, there were four centers, which were established soon after the outbreak was confirmed and located in three distant local hospitals and one neighboring hospital (Fig 1). In these centers, case-patients were identified, quarantined, and treated, and they were considered eligible for inclusion if they had laboratory-confirmed *V*. *cholerae* identified through conditional culture of rectal swabs and resided permanently in Ben Tre province. For this study, case-patients identified since the beginning of the outbreak were consecutively recruited until the target sample size was reached. Case-patients who were under cholera treatment were prospectively recruited through the four cholera treatment centers, while case-patients who had already been discharged from these health facilities were contacted and recruited at their homes.

For each case, four community-based controls [20] were selected and matched by commune, sex and 5-year age group to control for the potential confounding effects of these three factors. They were recruited at their homes on the same day as the interview of their matched case. Specifically, the control search began with random selection of four sub-communes in the area where the case resided by using Microsoft Excel. In each of these sub-communes, currently registered houses were numbered and one house was then chosen by hand drawing from the Vietnamese bingo game, a set containing 90 balls numbered 1–90. Interviewers subsequently accessed the selected house, in which all household members were primarily screened by sex and age to identify a potential control. Controls were further screened by interviewers to ensure that they had not had acute diarrhea in the month prior to the interview. If no person in that house met the inclusion criteria, the interviewers went to the next house to the left until a matched control was found. No control persons that we approached refused to participate in the present study and be interviewed.

Trained health-workers used a structured questionnaire to collect the study information during face-to-face interviews. This questionnaire was adapted from a cholera case investigation form previously developed and used in Ho Chi Minh City in 2008. For the present study, it was extended and included a section for controls. Risk variables included were based on reports in the literature [8, 21] and primary assessments conducted at the beginning of the outbreak. The questionnaire contained questions to elicit information on socio-demographic characteristics, exposure to people with diarrheal diseases, recent travel history, and detailed consumption of food and water. The logic and language of questions were tested locally in Ben Tre before being used to collect data. For children aged <6 years, interviews were conducted with their parents or guardians. Potential contaminated food sources were identified through dichotomous questions about the different types of food eaten, eating places, and cooking methods (e.g., well-cooked, stir fried, cooked rare, and raw) within five days before the onset of diarrhea for case-patients or five days before being interviewed for control persons. Participants were also asked about their sources and characteristics of water used for drinking, cooking, and bathing, and their drinking habits (e.g., the use of boiled water and drinking water with ice) via several dichotomous questions in the previous seven days. Clinical symptoms and health-seeking behaviors since the onset of diarrheal symptoms were also collected. Rectal swabs were collected from controls after completion of the interview.

### Laboratory analysis

During the outbreak, rectal swabs of people with acute diarrhea were directly placed in Cary Blair Transport Medium and transported daily to the Pasteur Institute, Ho Chi Minh City (PI-HCMC) for identification of *V*. *cholerae* using a standard testing protocol [22, 23]. All controls’ rectal swabs and environmental samples were stored at the Ben Tre Provincial Preventive Centre and transported fortnightly to the PI-HCMC for testing. Specifically, swab specimens were enriched in alkaline peptone water (Oxoid) and inoculated for 6–8 hours. We then cultured the bacterial solution obtained on thiosulfate citrate bile salts sucrose agar (Merck) and carried out biochemical and agglutination tests (Denka Seiken). We further used polymerase chain reaction (PCR) to amplify the *V*. *cholera* isolations from the swab culture, environmental samples, to identify the genomic region encoding the O1/O139 genotypes, and to detect cholera toxins and other toxin-specific genes, specifically *ctxA*, *ctxB*, *rtxC*, and *rstC*, using previously published primers [24–28] according to the PCR method described by Nguyen *et al*.[29]. A 9 μl of amplicon was separated by electrophoresis on a 1.5% agarose gel and visualized with an ultraviolet light on the Gel Doc System (Bio-Rad Laboratories). Test results were immediately reported to the attending physicians and the local preventive centers for personal treatment and prevention purposes, respectively.

#### Ethical statements

In Vietnam, the emergence of cholera is a significant health threat that requires a rapid public health response to protect the entire community from this severe, highly contagious diarrheal disease, and minimize any negative domino effects on population health. For this reason and in view of case-patients’ consent for cholera diagnosis and treatment at hospitals and the minimal risks to the subjects in this outbreak investigation, our request of a waiver of documentation of written informed consent for all participants was reviewed and approved by the local health authority in Ben Tre. A prepared sample verbal consent script was read to the participants. Verbal consents were obtained from all participants and documented through the use of a record of names kept in a secure and locked cabinet at the PI-HCMC. The outbreak investigation and study procedures have been reviewed and ratified by the PI-HCMC Institutional Review Board (reference number: 16/CN-HĐĐĐ).

### Statistical analysis

Statistical analysis was carried out using Stata version 14 (StataCrop LP, College Station, TX). Information about the participants in the case-control study were initially explored using descriptive statistics (including frequency and proportion for categorical variables and median and range for continuous variables), with comparisons between case-patients and controls made by McNemar’s chi-square tests. Children aged <6 years were classified as not having consumed a certain food or water if their parents or guardians reported that they did not consume it over the study period. To identify risk factors for cholera, conditional logistic regression with forward selection was used to estimate matched OR and 95% confidence intervals (CI) as per the method of Hosmer and his colleagues [30]. Initially, univariate analyses were employed to identify variables that were potential risk factors for cholera infection. Those variables with p<0.25, along with those which have been acknowledged to have biologic plausibility for increasing the risk of cholera, were considered for inclusion in the multivariable model. The analysis began by fitting the base model, which included cholera variable, participants’ age, and a variable that yielded the lowest corresponding p-value in univariate analyses. The variable with the next lowest p-value was progressively added to the model, and its contribution to model fit was tested by the log likelihood ratio test. If the test yielded a p-value less than 0.05, it was kept in the model. This process continued until the added variable made no significant improvement in model fit. As the level of education may behaviorally influence personal eating and drinking habits, we investigated potential effect modification by creating interaction terms between it and the other variables retained in the final model. In a sensitivity analysis, to accommodate missing data, we applied multiple imputation by chained equations, with an assumption of missingness *at random*. Ten imputed datasets were used with 1,000 iterations. We compared the risk factors found between the original and imputed data sets.

## Results

### The outbreak

**Fig 2:**
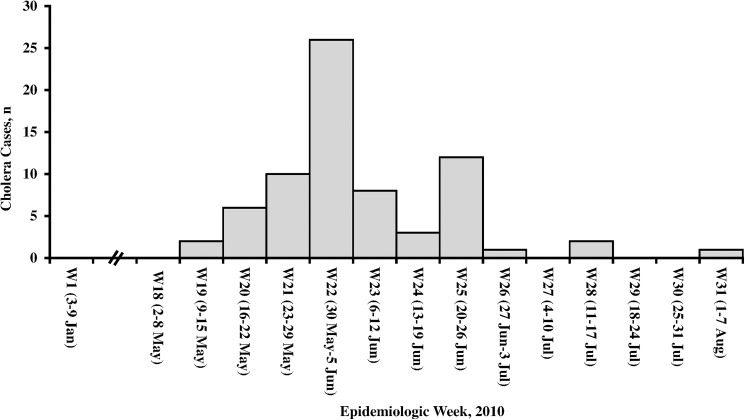
Distribution of culture confirmed cholera cases in Ben Tre, Vietnam, by epidemiologic week in 2010. The start and end dates of each epidemiologic week are shown.

From May 9 (epidemiologic week 19) through August 3 (epidemiologic week 31), 2010, 71 cases of cholera were identified in all towns of Ben Tre province. The area with the highest number of cases was Mo Cay (46 cases), followed by Giong Trom (12 cases) and Ba Tri (7 cases) (Fig 1). The number of cases continued to increase, peaked during May 30 to June 5 (epidemiologic week 22), and then gradually decreased over time (Fig 2). No case-patient died during the outbreak.

After confirmation of the outbreak on May 12, 2010, Ben Tre rapidly rolled-out its control activities in the entire province. It included measures to isolate and treat case-patients at four cholera treatment centers, clean and disinfect their houses with chloramine B, and provide chloramine B to enhance the use of chloramine-treated water among people living in the areas where the case-patients emerged. Case-patient’s close contacts were traced, given a single dose of prophylactic ciprofloxacin, and monitored their health. Education campaigns were additionally made to encourage people in the entire province to practice safe water, proper sanitation, and food safety. As reports of primary risk assessments suggested a potential link between drinking iced water and the illness, on June 11, 2010, the government of Ben Tre prohibited the manufacture, transportation, sale, or supply of commercial ice in the two most affected towns of Mo Cay and Giong Trom.

### Laboratory and environmental results

In total, only *V*. *cholerae* O1 Ogawa, El Tor biotype was isolated from 71cholera cases. The *ctxA*, *ctxB*, *rtxC*, and *rstC* genes were exhibited in 97%, 100%, 100%, and 97% of all isolates, suggesting a presence of altered El Tor strains that combine characteristics of classical and El Tor strains. Due to resource constraints, only 193 rectal swabs taken from 240 controls were tested for cholera. *V*. *cholerae* was not detected in any controls’ rectal swabs.

Two out of twenty five river water samples and one out of six wastewater samples were culture positive for *V*. *cholerae*. PCR analysis revealed the presence of *V*. *cholerae* O1 Ogawa carrying *ctxA* in all of these three positive-culture samples. *V*. *cholerae* was absent in two samples of drinking water, six samples of indoor water, and twenty-seven samples of fresh seafood.

### Case-control investigation

After excluding seven case-patients lost to follow-up and four case-patients whose disease occurred after the target sample size had been reached, a total of 60 case-patients occurring from May 9 through June 26 were consecutively included in a matched case-control study and matched to 240 controls. Forty case-patients were prospectively recruited through the cholera treatment centers and twenty case-patients were recruited at their homes. The case-patients were interviewed from June 4 through July 2, 2010. Nine case-patients and 32 control persons had missing data on at least one variable. Except for age, no significant discrepancies were recorded between the missing and non-missing subjects on variables (Table A in S1 File).

Demographic features and risk factors are listed in Table 1. Two thirds of subjects were females. Participants’ ages ranged from two months to 83 years for the case-patients and from one month to 87 years for the controls. For those aged six years or older, 73% of the case-patients and 57% of the controls reported having little education (primary education or illiterate).

**Table 1:** Characteristics of the matched case-control population.

Variable^a^	Case patients (N = 60) Frequency (%)	Controls (N = 240) Frequency (%)	p^b^
**Sociodemographic characteristics**			
Age (years), median (range)	45.5 (0.2–83)	42 (0.1–87)	-
Sex			-
Female	40 (67)	160 (67)	
Male	20 (33)	80 (33)	
Education level^c^			<0.001
Secondary or higher	15 (27)	94 (43)	
Primary education or illiterate	40 (73)	125 (57)	
**Hygiene**			
Facility for human fecal waste disposal			
Fishpond or river toilets	44 (73)	174 (73)	0.825
Outside latrine	2 (3)	3 (1)	0.227
Flush toilet	13 (22)	60 (25)	0.404
Main source of water close to a toilet	18 (30)	33 (14)	<0.001
Self-perceived changes in the color, odor, appearance and taste of water	9 (15)	20 (8)	0.029
Bathes with sedimented river water	31 (52)	71 (30)	<0.001
Brushes teeth/gargles with sedimented river water^c^	20 (33)	38 (16)	<0.001
**Drinking water**			
Drinks iced tea	13 (22)	6 (3)	<0.001
Drinks water with ice^c^			0.001
Never	11 (19)	75 (32)	
Sometimes, often or always	48 (81)	159 (68)	
Drinks boiled water^c^			<0.001
Always	21 (35)	136 (57)	
Sometimes, often or never	39 (65)	102 (43)	
Drinks sedimented river water	7 (12)	12 (5)	0.014
Drinks stored rainwater^c^	42 (70)	212 (89)	<0.001
Drinks bottled water	12 (20)	31 (13)	0.027
Drinks indoor tap water	2 (3)	9 (4)	1.000
**Food exposure/practices**			
Uses sedimented river water for cooking	25 (42)	51 (21)	<0.001
Uses stored rainwater for cooking	14 (23)	98 (41)	<0.001
Uses bottled water for cooking	0 (0)	2 (1)	0.500
Uses indoor tap water for cooking	4 (7)	28 (12)	0.058
Eats cooked seafood	37 (62)	194 (81)	<0.001
Eats raw seafood or seafood cooked rare	3 (5)	8 (3)	0.503
Eats steamed vegetables	7 (12)	74 (31)	<0.001
Eats raw vegetables	14 (23)	76 (32)	0.027
Eats fruits	7 (12)	17 (7)	0.090
**Others**			
Lives with people who had acute diarrhea	9 (15)	5 (2)	<0.001
Travels out of town^c^	21 (37)	48 (20)	<0.001

^a^Food consumption was collected for the 5 days before the onset of diarrhea for case-patients or before being interviewed for controls. Frequent water exposures and other risk behaviors and practices were collected in the previous seven days.

^b^P-values obtained from McNemar’s chi-square tests.

^c^Denominators may be lower than the total number because of missing data.

The median number of case-patient’s stools within a day prior to hospital admission was eight (range: 2–20), 55% had watery stools, 40% had abdominal pain, and 53% had vomiting. The mean time of admission to local hospitals was 0.7 days after the onset of the disease, and the mean time lag between case onset and enrolment of controls was 12 days among the case-patients.

Fishpond or river toilets were frequent in both the case-patient’s and control’s houses (73% and 73%, respectively). A flush toilet in house was reported in 22% of the case-patients and 25% of the controls (p = 0.404). Thirty percent of the case-patients reported that their main sources of water for use were close to a toilet, whereas only 14% of the controls had a similar situation (p <0.001). A self-report of changes in the color, odor, appearance and taste of water used was seen in 15% of the case-patients and 8% of the controls (p = 0.029).

Among the case-patients surveyed, 22% reported drinking iced tea within one week before the disease onset, compared with 3% of the controls in the week before being interviewed (p <0.001). A higher percentage of the case-patients than the controls consumed water with ice (81% vs. 68%, p = 0.001), unboiled water (65% vs. 43%, p <0.001), and sedimented river water (12% vs. 5%, p = 0.014). Up to 52%, 33%, and 42% of the case-patients used sedimented river water as the source of water for bathing, brushing teeth/gargling, and cooking, respectively. These levels were nearly twice as high as those for the controls (30%, 16%, and 21%, respectively; all p-values <0.001). The case-patients were less likely to use stored rainwater for both drinking (70% vs. 89%, p <0.001) and cooking (23% vs. 41%, p <0.001), but they were more likely to drink bottled water (20% vs. 13%, p = 0.027) than the controls. The use of indoor tap water for both drinking and cooking was rarely seen in this sample.

The case-patients were less likely to eat cooked seafood (62% vs. 81%, p<0.001), steamed vegetables (12% vs. 31%, p <0.001) and raw vegetables (23% vs. 32%, p = 0.027) in comparison with the controls.

While only 2% of the controls were living with people who had acute diarrhea, 15% of the case-patients did so (p <0.001). The case-patients had a greater percentage of travel out of town in the week prior to the disease onset than the controls (37% vs. 20%, p <0.001).

Risk factors for cholera infection identified through the multivariable conditional logistic regression are summarized in Table 2. Individuals who reported drinking iced tea (adjusted OR (aOR) = 8.40, 95% CI: 1.84–39.25), not always boiling drinking water (aOR = 2.62, 95% CI: 1.03–6.67), living with people who had acute diarrhea (aOR = 13.72, 95% CI: 2.77–67.97), having the main household sources of water for use close to a toilet (aOR = 4.36, 95% CI: 1.37–13.88), and having little or no education (aOR = 4.89, 95% CI: 1.18–20.19) were significantly associated with an elevated risk of cholera. The risk of cholera was lower among persons who drank stored rainwater (aOR = 0.17, 95% CI: 0.04–0.63), ate cooked seafood (aOR = 0.27, 95% CI: 0.10–0.73), and ate steamed vegetables (aOR = 0.22, 95% CI: 0.07–0.70). No significant interactions were recorded throughout the analysis. Only the level of education become insignificant in another regression analysis based on a multiple imputation approach (Table B in S1 File).

**Table 2:** Conditional logistic regression analysis of cholera risk factors in Ben Tre, southern Vietnam, 2010.

Variable	Univariate analysis		Multivariate analysis^a^	
	Crude OR (95% CI)	p	Adjusted OR (95% CI)	p
**Sociodemographic factors**				
Age (years)^b^	1.07 (0.99–1.15)	0.074	1.07 (0.97–1.17)	0.187
Education level				
Secondary or higher	ref		ref	
Primary education or illiterate	3.49 (1.32–9.22)	0.012	4.89 (1.18–20.19)	0.028
**Hygiene**				
Flush toilet in house	0.82 (0.40–1.66)	0.574	n/a^c^	n/a^c^
Main source of water close to a toilet	3.60 (1.62–7.99)	0.002	4.36 (1.37–13.88)	0.013
Self-perceived changes in the color, odor, appearance and taste of water	1.99 (0.84–4.71)	0.119	-	-
Bathes with sedimented river water	2.84 (1.52–5.31)	0.001	2.33 (0.93–5.82)	0.070
Brushes teeth/gargles with sedimented river water	2.80 (1.44–5.48)	0.003	-	-
**Drinking water**				
Drinks iced tea	11.89 (3.85–36.70)	<0.001	8.40 (1.84–39.25)	0.006
Drinks water with ice				
Never	ref			
Sometimes, often or always	2.11 (1.01–4.40)	0.046	-	-
Drinks boiled water				
Always	ref		ref	
Sometimes, often or never	2.71 (1.44–5.09)	0.002	2.62 (1.03–6.67)	0.044
Drinks sedimented river water	2.58 (0.94–7.06)	0.064	-	-
Drinks stored rainwater	0.25 (0.12–0.53)	<0.001	0.17 (0.04–0.63)	0.008
Drinks bottled water	1.78 (0.81–3.90)	0.149	-	-
Drinks indoor tap water	0.89 (0.19–4.11)	0.880	n/a^c^	n/a^c^
**Food exposure/practices**				
Uses sedimented river water for cooking	2.93 (1.54–5.58)	0.001	-	-
Uses stored rainwater for cooking	0.39 (0.20–0.79)	0.008	-	-
Uses indoor tap water for cooking	0.40 (0.11–1.54)	0.184	-	-
Eats cooked seafood	0.37 (0.20–0.70)	0.002	0.27 (0.10–0.73)	0.009
Eats raw seafood or seafood cooked rare	1.50 (0.40–5.65)	0.549	n/a^c^	n/a^c^
Eats steamed vegetables	0.26 (0.11–0.61)	0.002	0.22 (0.07–0.70)	0.011
Eats raw vegetables	0.64 (0.32–1.26)	0.195	-	-
Eats fruits	1.97 (0.69–5.61)	0.207	-	-
**Others**				
Lives with people who had acute diarrhea	7.20 (2.41–21.48)	<0.001	13.72 (2.77–67.97)	0.001
Travels out of town	2.57 (1.26–5.23)	0.009	-	-

Reference is the absence of a characteristic, unless otherwise specified. CI, confidence interval; OR, odds ratio.

^a^The analysis was based on 265 observations with complete data. The estimated pseudo-R-squared of the final model was 44.9%.

^b^Age was modeled as a continuous variable.

^c^This variable was not selected for multivariate analysis due to a corresponding p-value above 0.25 in univariate analysis.

## Discussion

In this investigation of the 2010 Ben Tre cholera outbreak, most risk factors identified have been previously described and associated with exposure to unsafe water (such as, not always drinking boiled water, main source of water close to toilet) or with proximity with another possible case who had acute diarrhea. Our results revealed a significantly increased risk of cholera among individuals who reported drinking iced tea in the week before the onset of their illness. We also observed substantial cholera risk reductions in some subpopulations, specifically those who drank stored rainwater and ate cooked seafood or steamed vegetables.

Our study suggested that the spread of cholera in Ben Tre was quite considerable, but not nearly as high as had been seen in Hanoi [11] and other Southeast Asian settings [31, 32]. Early outbreak detection, community-engaged health promotion, large-scale distribution of chloramine B to treat surface water, its low population density (an estimated 533 people/km^2^), and a prohibition of ice block production business during the outbreak could have contributed to the relatively smaller size of the outbreak in Ben Tre province. In agreement with previous studies [14, 26, 32, 33], we found evidence that *V*. *cholerae* O1, altered El Tor was prevalent among the case-patients. There were no reported deaths attributable to any strains of cholera among the case-patients in this outbreak. This finding could help to address the previously reported clinical concern that the spread of these strains with increased severity would lead to more cholera deaths in some areas of the world [34].

We know of no previous studies that have reported increased risk of infection specifically associated with iced tea consumption. Locally, iced tea is made by adding cooled boiled tea to a glass or bottle of ice. Ice is typically bought from street vendors rather than prepared at home in rural or semi-rural areas of Vietnam because less than one-eighth (12%) of rural households own a refrigerator [35]. A previous hospital-based case-control study conducted in Thailand demonstrated that consumption of ice was significantly associated with increased risk of cholera in a bivariate analysis but was not significant in the multivariable model [8]. In our outbreak investigation, we were not able to obtain commercial and household ice for testing for the presence of *V*. *cholerae* in ice. We were therefore unable to ascertain the underlying microbiological mechanism leading to the association between drinking iced tea and cholera. A previous study, conducted in Jakarta, Indonesia, showed that a large percentage of ice (35%) and beverages (24%) were contaminated with *V*. *cholerae* [36]. In Vietnam where untreated wells and surface water (i.e. river water) have been commonly used for making commercial ice, it is possible for *V*. *cholerae* to be introduced into commercial ice, and such contamination could have triggered this resurgence of cholera in Ben Tre province. The observation that the decline in the weekly reported number of cholera was seen after the local government prohibited on the manufacture, transportation, sale, or supply of commercial ice in the outbreak area supports this hypothesis. In future outbreaks, further epidemiological and microbiological investigation of the association between commercial ice and cholera is warranted. The microbiological quality of the water used at ice manufacturing plants should be tested regularly.

Our other risk factors for cholera included living with people who had acute diarrheal illness and not always boiling drinking water. These are well-described predictors of cholera in endemic countries [8, 37]. The association between having the main source of water for use being close to the toilets indicates that pollution of drinking water sources by contaminated feces remains a problem in Vietnam and could lead to further spread of disease in future cholera outbreaks [38]. As expected, we found that drinking stored rainwater, eating cooked seafood, and eating steamed vegetables were protective against cholera, and these results are consistent with findings from several previous studies [21, 39]. Local public health authorities should rapidly roll out a response that involves appropriate case management and greater promotion of proper sanitation, safe water, and food safety in the event of a cholera outbreak [40].

Our observation that individuals who reported little or no education were at a higher risk of cholera is consistent with the results of a previous case-control study in Harare City, Zimbabwe in which having attained less than secondary education was found to be a risk factor for cholera [41]. Poor education is part of the cycle of poverty, which includes crowded living conditions, malnourishment, poor household sanitation and personal hygiene practices [42], which are well-known to be important risk factors for cholera [43].

This study has several limitations. First, the number of cholera cases reported herein might not have reflected the actual burden of cholera in Ben Tre province. The vast majority of people infected with *V*. *cholerae* are asymptomatic or mildly symptomatic, and thus might not seek healthcare services [44]. Second, detection of *V*. *cholerae* in some water samples obtained only from Mo Cay and Giong Trom should be interpreted with caution, because the degree to which these samples are representative of environmental sources in other towns throughout the province where case-patients occurred is unknown. Third, selection of controls from the commune where the case-patients resided can limit exploration of some important geographical and cultural risk factors, such as exposure to river water, due to similar exposures between the controls and the case-patients. Fourth, reduced recall of information may have occurred among case-patients recruited and interviewed late. To reduce the need to recall distant exposures among both the case-patients and controls, study questions were specific to food/water and location. Fifth, about 20% of controls’ rectal swabs were not tested for cholera, potentially misclassifying case-patients as controls. However, such misclassification was unlikely as 80% of controls’ rectal swabs tested were negative for *V*. *cholerae*. Sixth, as with other behavioral studies that depend entirely on face-to-face interviews, this study may under-report some important risks, such as food and personal hygiene practices. To reduce this bias, we selected well-trained, experienced, and unprejudiced interviewers after the interview training. Lastly, our study may not have enough power to detect rare risk factors for cholera.

Despite these limitations, this present study has important implications for Vietnam’s cholera responses. This emergence of cholera due to *V*. *cholerae* O1, altered El Tor emphasizes the need for increased efforts to prevent the spread of cholera in southern Vietnam. Along with traditional approaches that focus on enhancement of safe water, sanitation, and food safety, combined with periodic provision of oral cholera vaccines, a water quality monitoring system at ice-making plants should be established. It is vital to ensure the quality of the water supply, reduce the introduction of *V*. *cholerae* into ice, and subsequently lower the risk of cholera in Vietnam, a tropical setting where consumption of iced drinks is common.

## Supporting information

S1 Checklist STROBE Checklist. (PDF)

S1 File Supplementary Materials. Characteristics of the sample with and without missing data (**Table A**). Cholera risk factors in Ben Tre, southern Vietnam, 2010 (**Table B**).
(DOCX)
