# Supplementary material for: Cholera returns to southern Vietnam in an outbreak associated with consuming unsafe water through iced tea: A matched case-control study
Source: PLoS Negl Trop Dis. 2017 Apr 13;11(4):e0005490. doi: 10.1371/journal.pntd.0005490 (PMC5390973; doi:10.1371/journal.pntd.0005490)
Supplement: S1 File — Characteristics of the sample with and without missing data (Table A). Cholera risk factors in Ben Tre, southern Vietnam, 2010 (Table B). (DOCX) [file pntd.0005490.s002.docx]

## S1.File. Supplementary Materials

**Table A. Characteristics of the sample with and without missing data**

| **Variable** | | **People without missing data (N=259)** | | **People who had missing data**  **on at least one variable** | | | **p-value** |
| --- | --- | --- | --- | --- | --- | --- | --- |
|  |  | **n** | **%** | **N** | **n** | **%** |  |
| **Sociodemographics** | |  |  |  |  |  |  |
| Age (years), median (range) | | 44 (7 – 85) | | 30 (0.1 – 87) | | | <0.001 |
| Sex | Female | 173 | 67 | 41 | 27 | 66 | 0.905 |
|  | Male | 86 | 33 | 41 | 14 | 34 |  |
|  | |  |  |  |  |  |  |
| Education level^a^ | Secondary or higher | 103 | 40 | 15 | 6 | 40 | 0.986 |
|  | Primary education/ illiterate | 156 | 60 | 15 | 9 | 60 |  |
| **Hygiene** |  |  |  |  |  |  |  |
| Bathes with sedimented river water | No | 174 | 67 | 41 | 24 | 59 | 0.278 |
|  | Yes | 85 | 33 | 41 | 17 | 41 |  |
| Brushes teeth/gargles with sedimented river water^a^ | No | 211 | 81 | 40 | 30 | 75 | 0.336 |
|  | Yes | 48 | 19 | 40 | 10 | 25 |  |
| Facility for human fecal waste disposal | Fishpond or river toilet | 194 | 74 | 41 | 24 | 58.5 | 0.029 |
|  | Outside latrine | 4 | 2 | 41 | 1 | 2 | 0.523 |
|  | Flush toilet | 61 | 24 | 41 | 12 | 29 | 0.437 |
| Main source of water close to a toilet | No | 211 | 81 | 41 | 38 | 93 | 0.076 |
|  | Yes | 48 | 19 | 41 | 3 | 7 |  |
| Self-perceived changes in the color, odor, appearance and taste of water | No | 234 | 90 | 41 | 37 | 90 | 0.983 |
|  | Yes | 25 | 10 | 41 | 4 | 10 |  |
| **Drinking water** |  |  |  |  |  |  |  |
| Drinks iced tea | No | 244 | 94 | 41 | 37 | 90 | 0.308 |
|  | Yes | 15 | 6 | 41 | 4 | 8 |  |
| Drinks water with ice^a^ | Never | 76 | 29 | 34 | 10 | 29 | 0.993 |
|  | Sometimes, often or always | 183 | 71 | 34 | 24 | 71 |  |
| Drinks boiled water^a^ | Always | 137 | 53 | 39 | 20 | 51 | 0.851 |
|  | Sometimes, often or never | 122 | 47 | 39 | 19 | 49 |  |
| Drinks bottled water | No | 225 | 87 | 41 | 32 | 78 | 0.134 |
|  | Yes | 34 | 13 | 41 | 9 | 22 |  |
| Drinks indoor tap water | No | 251 | 97 | 41 | 38 | 93 | 0.179 |
|  | Yes | 8 | 3 | 41 | 3 | 7 |  |
| Drinks stored rainwater | No | 37 | 14 | 39 | 7 | 18 | 0.548 |
|  | Yes | 222 | 86 | 39 | 32 | 82 |  |
| Drinks sedimented river water | No | 241 | 93 | 41 | 40 | 98 | 0.488 |
|  | Yes | 18 | 7 | 41 | 1 | 2 |  |
| **Food exposure/practices** |  |  |  |  |  |  |  |
| Uses bottled water for cooking | No | 257 | 99 | 41 | 41 | 100 | 1.000 |
|  | Yes | 2 | 1 | 41 | 0 | 0 |  |
| Uses indoor tap water for cooking | No | 231 | 89 | 41 | 37 | 90 | 1.000 |
|  | Yes | 28 | 11 | 41 | 4 | 8 |  |
| Uses stored rainwater for cooking | No | 166 | 64 | 41 | 22 | 54 | 0.199 |
|  | Yes | 93 | 36 | 41 | 19 | 46 |  |
| Uses sedimented river water for cooking | No | 195 | 75 | 41 | 29 | 71 | 0.533 |
|  | Yes | 64 | 25 | 41 | 12 | 29 |  |
| Eats cooked seafood | No | 58 | 22 | 41 | 11 | 27 | 0.531 |
|  | Yes | 201 | 78 | 41 | 30 | 73 |  |
| Eats raw seafood or seafood cooked rare | No | 249 | 96 | 41 | 40 | 98 | 1.000 |
|  | Yes | 10 | 4 | 41 | 1 | 2 |  |
| Eats steamed vegetables | No | 187 | 72 | 41 | 32 | 78 | 0.433 |
|  | Yes | 72 | 28 | 41 | 9 | 22 |  |
| Eats raw vegetables | No | 177 | 68 | 41 | 33 | 80 | 0.115 |
|  | Yes | 82 | 32 | 41 | 8 | 20 |  |
| Eats fruits | No | 237 | 92 | 41 | 39 | 95 | 0.551 |
|  | Yes | 22 | 8 | 41 | 2 | 5 |  |
| **Others** | |  |  |  |  |  |  |
| Lives with people who had acute diarrhea | No | 247 | 95 | 41 | 39 | 95 | 0.945 |
|  | Yes | 12 | 5 | 41 | 2 | 5 |  |
| Travel out of town^a^ | No | 199 | 77 | 34 | 25 | 74 | 0.669 |
|  | Yes | 60 | 23 | 34 | 9 | 26 |  |

^a^Variables with missing data.

**Table B. Cholera risk factors in Ben Tre, southern Vietnam, 2010.**

| **Variable** | | **Multivariate analysis  (multiple imputation)** | |
| --- | --- | --- | --- |
|  |  | **aOR (95% CI)** | **p-value** |
| Age (years) | | 1.07 (0.98 – 1.17) | 0.142 |
| Education level^a^ | Secondary or higher | ref |  |
|  | Primary education or illiterate | 3.47 (0.89 – 13.49) | 0.073 |
| Lives with people who had acute diarrhea | No | ref |  |
|  | Yes | 8.33 (1.84-37.22) | 0.006 |
| Main source of water close to a toilet | No | ref |  |
|  | Yes | 4.89 (1.61 – 14.83) | 0.005 |
| Bathes with sedimented river water | No | ref |  |
|  | Yes | 2.08 (0.88 – 4.90) | 0.095 |
| Drinks iced tea | No | ref |  |
|  | Yes | 8.71 (2.04 – 37.21) | 0.003 |
| Drinks boiled water^a^ | Always | ref |  |
|  | Sometimes, often or never | 2.91 (1.21 – 7.01) | 0.017 |
| Drinks stored rainwater | No | ref |  |
|  | Yes | 0.18 (0.05 – 0.64) | 0.008 |
| Eats cooked seafood | No | ref |  |
|  | Yes | 0.27 (0.11 – 0.67) | 0.005 |
| Eats steamed vegetables | No | ref |  |
|  | Yes | 0.29 (0.07 – 0.86) | 0.025 |
